# The significance of Meckel's scan in pediatric gastrointestinal bleeding cases: A case report

**DOI:** 10.1016/j.radcr.2024.03.052

**Published:** 2024-04-20

**Authors:** Damayanti Sekarsari, Ella Putri Saptari, Mohamad Yanuar Amal, Quinta Febryani Handoyono, Alvita Dewi Siswoyo, Sastiono, Hanifah Oswari, Ening Krisnuhoni

**Affiliations:** a Division of Pediatric Radiology, Department of Radiology, Faculty of Medicine, University of Indonesia, Dr. Cipto Mangunkusumo General Hospital, Salemba Raya No. 4, Kenari, Senen, Central Jakarta, DKI Jakarta, Indonesia; b Department of Radiology, Faculty of Medicine, University of Indonesia, Dr. Cipto Mangunkusumo General Hospital, Salemba Raya No. 4, Kenari, Senen, Central Jakarta, DKI Jakarta, Indonesia; c Department of Nuclear Medicine, Faculty of Medicine, University of Indonesia, Dr. Cipto Mangunkusumo General Hospital, Salemba Raya No. 4, Kenari, Senen, Central Jakarta, DKI Jakarta, Indonesia; d Division of Pediatric Surgery, Department of Surgery, Faculty of Medicine, University of Indonesia, Dr. Cipto Mangunkusumo General Hospital, Salemba Raya No. 4, Kenari, Senen, Central Jakarta, DKI Jakarta, Indonesia; e Division of Gastrohepatology, Department of Pediatrics, Faculty of Medicine, University of Indonesia, Dr. Cipto Mangunkusumo General Hospital, Salemba Raya No. 4, Kenari, Senen, Central Jakarta, DKI Jakarta, Indonesia; f Department of Anatomic Pathology, Faculty of Medicine, University of Indonesia, Dr. Cipto Mangunkusumo General Hospital, Salemba Raya No. 4, Kenari, Senen, Central Jakarta, DKI Jakarta, Indonesia

**Keywords:** Pediatric gastrointestinal bleeding, Meckel's diverticulum, Tc-99m pertechnetate scintigraphy, SPECT/CT

## Abstract

Meckel's diverticulum is the most prevalent congenital anomaly of the gastrointestinal tract, identified in 2% of the population according to autopsy studies. Most patients remain asymptomatic throughout their lives and are typically diagnosed when complications arise. The diagnosis can be challenging, but imaging is crucial for promptly identifying and distinguishing it from other conditions that have similar clinical manifestations. A 13-year-old male was admitted with a 5-day history of rectal bleeding. The patient continued to experience painless gastrointestinal bleeding, indicating the performance of a Tc-99m pertechnetate scintigraphy or Meckel's scan. Planar images revealed focal uptake within the right hemiabdomen, suggestive of the presence of a Meckel's diverticulum. Subsequent laparotomy surgery confirmed the presence of a Meckel's diverticulum located 50 cm from the ileocecal valve. Histopathological examination of the resected specimen confirmed Meckel's diverticulum with ectopic gastric mucosa. This patient with Meckel's diverticulum exhibited minimal abdominal symptoms, and there were no other complications such as intussusception, which could lead to bowel obstruction. Technetium-99m pertechnetate scintigraphy is a common method for evaluating children with unexplained gastrointestinal tract bleeding. SPECT/CT fusion imaging enables the simultaneous fusion of functional and anatomical information, preventing false-negative scintigraphy examinations. Its capability to precisely localize activity in abnormal structures contributes to accurate scan interpretation. Complications of Meckel's diverticulum are uncommon and pose a diagnostic challenge. Through comprehensive history-taking, physical examination, and nuclear imaging, the diagnosis can be identified, and surgical intervention can be performed to achieve the best possible outcome for the patient.

## Background

Meckel's diverticulum is a common congenital anomaly in the development of the gastrointestinal system [1]. This condition arises from the failure of closure of the omphalomesenteric (vitelline) duct, connecting the midgut in embryos to the yolk sac [1]. It exhibits the characteristic “rule of 2,” which are: occurring in 2% of the population, with a male-to-female ratio of 2:1, and an incidence rate of 2% for symptomatic Meckel's diverticulum [2]. Symptoms typically manifest before the age of 2, with the diverticulum located approximately 2 feet from the ileocecal valve, measuring around 2 inches in length, and featuring 2 types of heterotopic tissues [2]. Usually asymptomatic, Meckel's diverticulum is incidentally discovered, with reported complication risks ranging from 4% to 40% [3]. Only 4%-25% of individuals with Meckel's diverticulum exhibit symptoms, usually occurring before the age of 10 [4]. Histologically, heterotopic gastric or pancreatic mucosa is often found. Common complications include gastrointestinal bleeding from mucosal inflammation, small bowel obstruction, and diverticulitis, with gastrointestinal bleeding being a frequent complication [5,6]. Radiology plays a crucial role in detecting Meckel's diverticulum and its complications [3]. Diagnosing Meckel's diverticulum is challenging, and imaging is pivotal in confirming the diagnosis and distinguishing it from other conditions with similar clinical presentations [6].

Multiple imaging techniques can be employed for the diagnosis of Meckel's diverticulum. Conventional radiography provides limited information but may reveal complications such as enteroliths, small bowel obstruction, or the presence of air-fluid levels in the diverticulum. GI studies, including follow-through, enteroclysis, or contrast enema, have largely been replaced by other techniques in patients with acute symptoms. GI studies often fail to visualize the diverticulum due to its small ostium and food-filled content. In CT scans, uncomplicated Meckel's diverticulum is challenging to differentiate from normal small bowel [3,7]. Gastrointestinal bleeding is an indication for nuclear studies like Meckel's scan or Meckel scintigraphy using Tc-99m pertechnetate to detect heterotopic gastric mucosa causing ulcers in the surrounding mucosa. The fusion of single-photon emission computed tomography/computed tomography (SPECT/CT) with a gamma camera represents an innovative technology providing the integration of functional and anatomical information, reducing the probability of false negatives [4]. This case report highlights the role of SPECT/CT in diagnosing gastrointestinal bleeding due to Meckel's diverticulum, aiding in determining patient management and surgical interventions, ultimately leading to a favorable outcome for the patient.

## Case report

A 13-year-old boy was brought by his parents with a 5-day history of intermittent rectal bleeding and malaise. The stool was described as dark red, and other symptoms such as abdominal pain, nausea and vomiting, fever, or bleeding in other sites were not present. Any history of trauma, allergy, other chronic diseases, and family history of similar symptoms were denied. The patient had previously received treatment for the same symptom in 2019 and was diagnosed with intestinal inflammation. From physical examination, the vital signs were stable, conjunctivae were anemic, abdomen was not distended, no abdominal pain on palpation, no organomegaly was found, and other physical examinations were within normal limits. Laboratory examination was performed and revealed that the patient was anemic with Hb of 8.1 g/dL, Ht of 23.1%, erythrocyte of 2.86 million/µL, MCV of 80.8 fL, MCHC of 35.1 g/dL, thrombocyte of 406 thousand/µL, and leukocyte of 14.66 thousand/µL. Subsequently, imaging was arranged to thoroughly assess the patient's condition and establish the diagnosis.

**Fig. 1:**
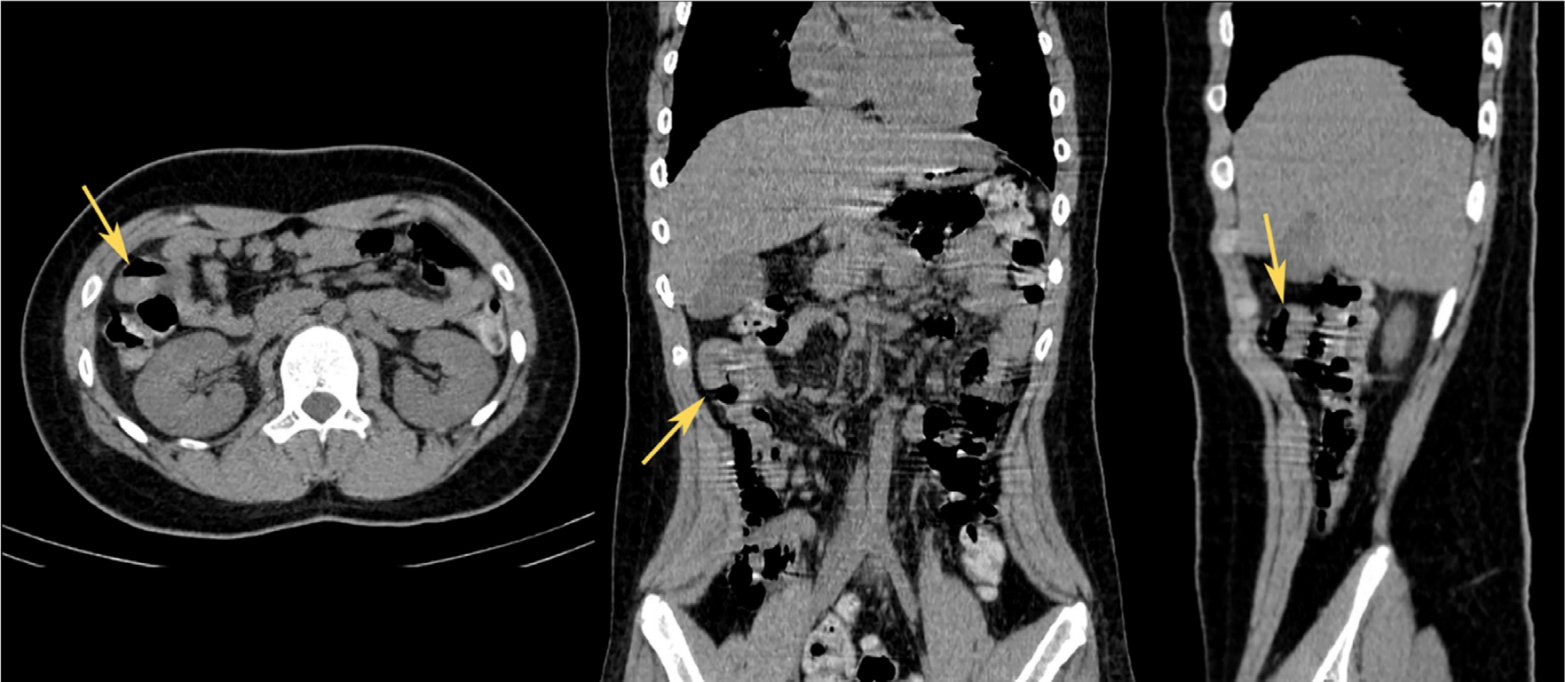
A non-contrast CT scan with axial, coronal, and sagittal images only showed a thickened intestinal mucosa (marked by arrows).

**Fig. 2:**
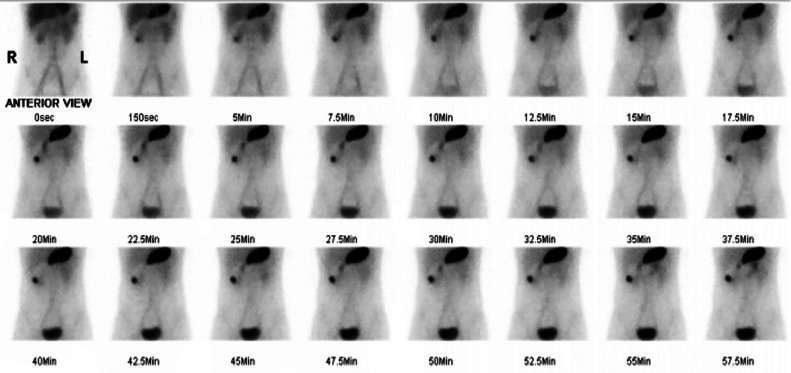
Tc-99m pertechnetate scintigraphy or Meckel's scan identified a focal uptake within the right upper hemiabdomen which coincides with normal radiotracer uptake in the stomach in sequential anterior planar images acquired over 60 minutes (2.5 min/frame).

**Fig. 3:**
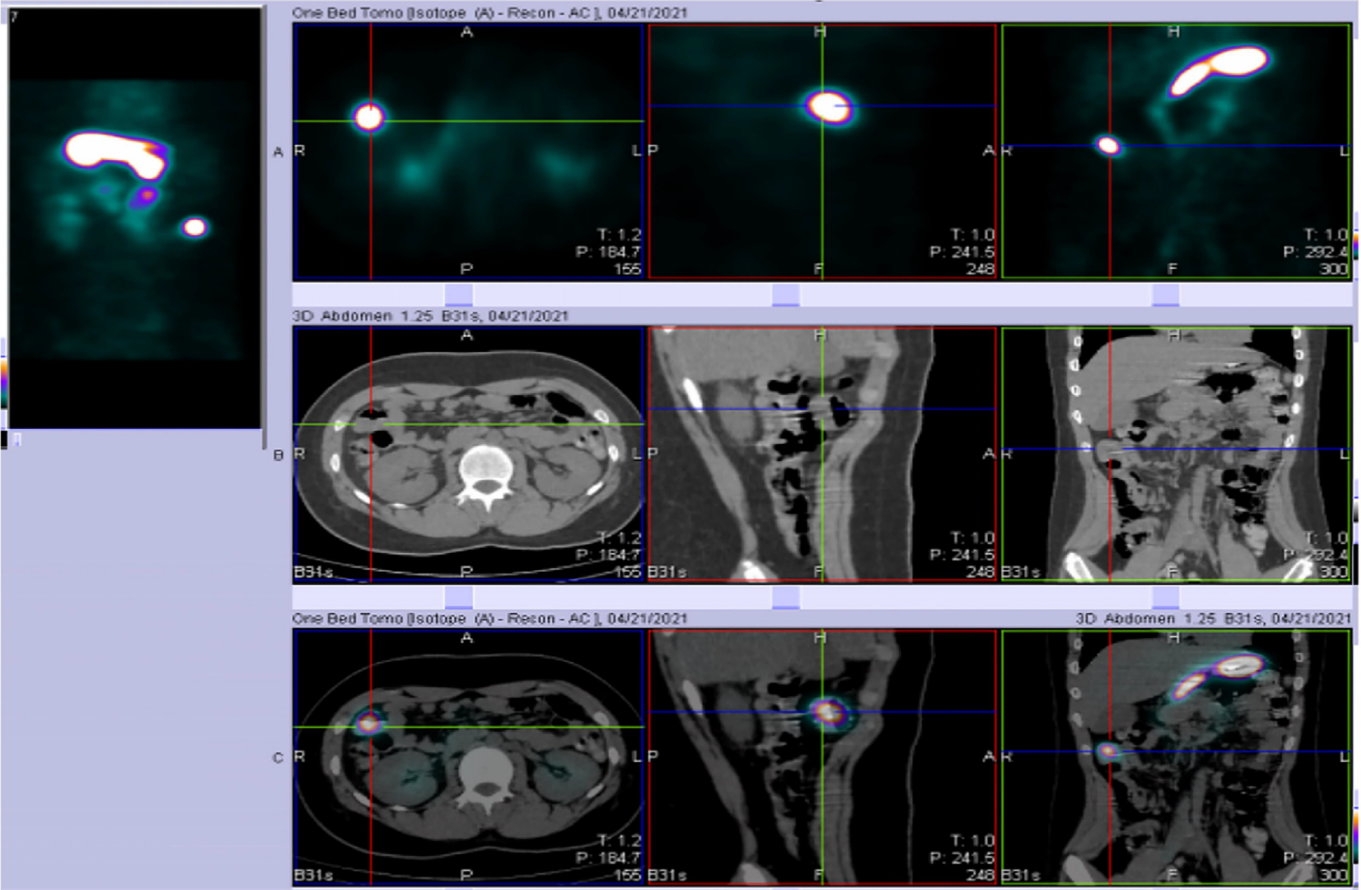
SPECT/CT fusion imaging revealed a focal radiotracer accumulation within the right upper hemiabdomen localized to an apparent blind-ending loop of bowel in the small intestine.

Non-contrast CT scan was performed to determine the cause of the bleeding, with axial, coronal, and sagittal images only showed a thickened intestinal mucosa (shown by arrows in Fig. 1). Unexplained GI bleeding in pediatric patients raised our suspicion of a symptomatic Meckel's diverticulum, with GI bleeding is one of the more frequent complications. The patient then underwent Meckel's scan at our facility to further evaluate the diagnosis, utilizing Tc-99m pertechnetate intravenous radioisotope of 10 mCi dynamically and serially in static phases, the result is shown in Fig. 2. Physiological activity in the gastric region was observed from the first minute, peaking at the 10th minute, and persisting until the end of the examination in both dynamic and static imaging. Concurrent with gastric activity, pathological activity beyond the stomach appeared in the right upper hemiabdomen projection during the dynamic phase and static serials from the first minute, progressively increasing (activity retention) until the conclusion of the examination. Physiological activity by the bowel was visible at the 7.5-minute mark, progressively increasing until the end of the examination. SPECT/CT confirmation revealed pathological activity capture in the right upper hemiabdomen at the level of the midpole of the right kidney, projecting to the ileum, originating from isodense tissue (consistent with the ileum's intestinal mucosa), suggestive of Meckel's diverticulum in the right upper hemiabdomen projection to the ileum (Fig. 3).

**Fig. 4:**
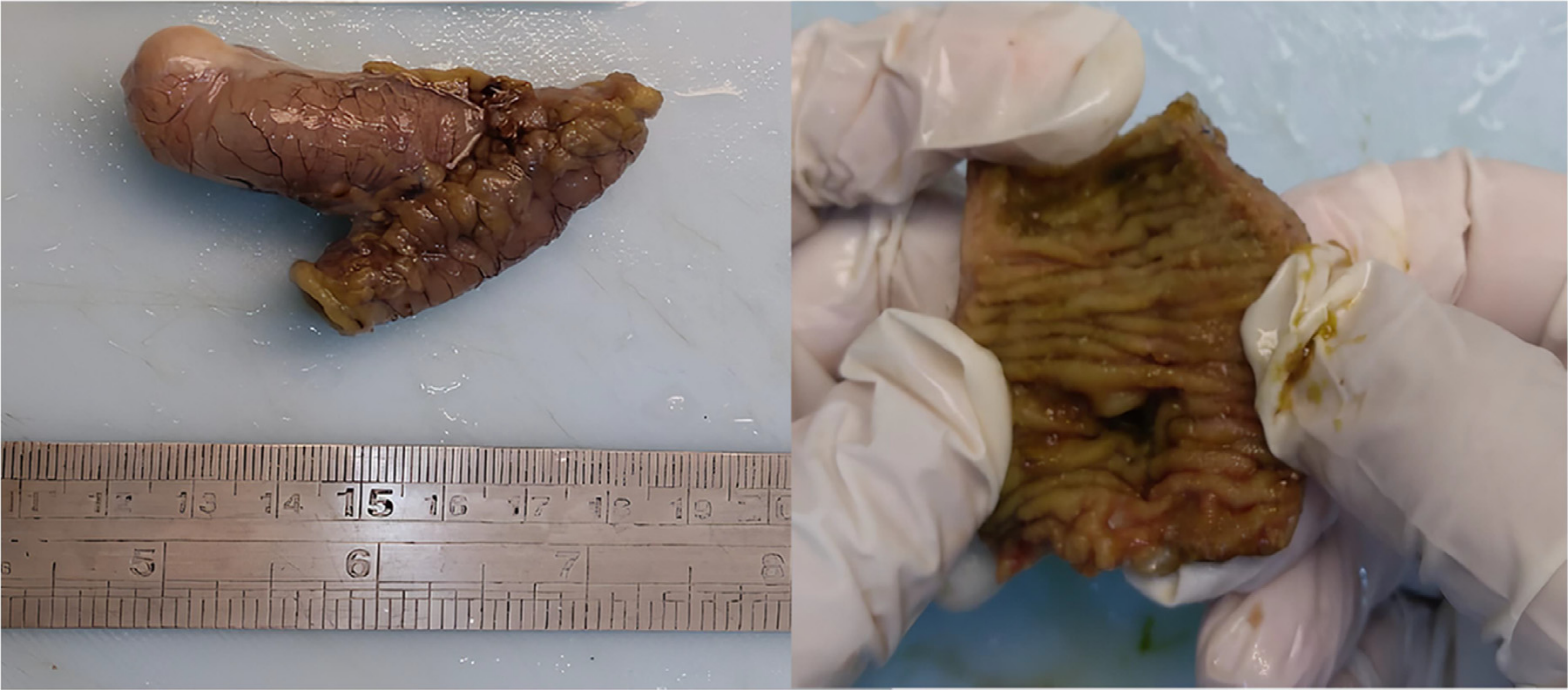
Surgical intervention confirmed the presence of a Meckel's diverticulum 50 cm from the ileocecal valve. Surgical specimen included of a 5 cm segment of the ileum with an out-pouching presented as polypoid mass that measured 4 cm long and 1 cm in diameter.

**Fig. 5:**
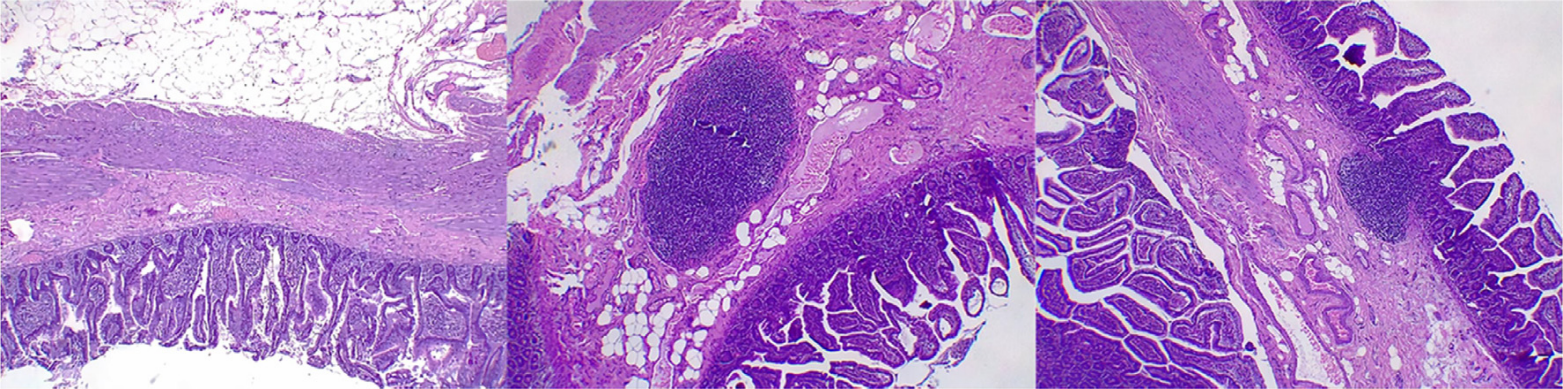
Histopathology examination revealed the mucosa was composed of an ectopic gastric mucosa. The lamina propria demonstrated mild to moderate chronic inflammatory cell infiltration, forming lymphoid follicles. The mucosal and serosal layers appeared edematous with congested blood vessels. The muscularis propria displayed fasciculations. The histological examination confirms the diagnosis of Meckel's diverticulum with ectopic gastric mucosa.

Subsequently, the patient underwent laparotomy for ileo-ileal resection and anastomosis, revealing a pouch protruding into the extraluminal space identified approximately 50 cm from the ileocaecal valve (Fig. 4). The specimen was sent to the pathology department for histopathological analysis. One piece of intestinal tissue measuring 5.5 cm in length, containing 2 lumens measuring 0.5 cm and 1.0 cm, with a protruding area of 4 cm in length, was received. Microscopic examination revealed an enteric portion entering the mucosa, measuring 0.5 cm in diameter, 4 cm in length, positioned 2.5 cm from the first lumen and 2 cm from the second lumen. No erosions, perforations, or tumor masses were identified. The mucosa exhibited an ectopic gastric mucosa. The lamina propria demonstrated mild to moderate chronic inflammatory cell infiltration, forming lymphoid follicles. The mucosal and serosal layers appeared edematous with congested blood vessels. The muscularis propria displayed fasciculations. The histological examination confirms the diagnosis of Meckel's diverticulum with ectopic gastric mucosa, as shown in Fig. 5.

Following a 6-day postoperative care period, the patient reported no complaints. Subsequently, the patient returned for a follow-up in good condition for postoperative dressing replacement.

## Discussion

Meckel's diverticulum occurs when the omphalomesenteric duct or vitelline duct, which connects the yolk sac to the midgut through the umbilical cord, fails to obliterate properly. Typically, this duct undergoes obliteration during gestational weeks 5-8. Non-closure of the duct leads to diverticulum formation (in 90% of cases), omphalomesenteric fistula, enterocyst, or fibrous band. The diverticulum arises from the antimesenteric border of the small intestine. It measures approximately 5 cm in length and 2 cm in diameter, with vascularization from the omphalomesenteric artery [3,8]. Meckel's diverticulum is considered the most common congenital anomaly of the gastrointestinal tract [1]. It is typically diagnosed based on clinical presentation, imaging studies, and occasionally, intraoperative findings. The presence of a heterotopic mucosa within the diverticulum, is considered a hallmark of Meckel's diverticulum. Other diagnostic criteria include the location of the diverticulum within 100 cm of the ileocecal valve, its typical size ranging from 2 to 4 cm, and the presence of a connection to the antimesenteric border of the ileum [3].

While many Meckel's diverticulum remains asymptomatic throughout life, they can lead to various complications. In clinical practice, despite its prevalence, diagnosing Meckel's diverticulum can be challenging due to its symptoms and even complications, which often mimic those of other gastrointestinal disorders. Therefore, a high index of suspicion is required, especially in cases of unexplained GI bleeding in pediatric patients. Differential diagnoses may include appendicitis, ileal, or colonic diverticulitis, regional enteritis, colitis, enteric duplication cysts, and intussusception [6]. It is essential to understand the prevalence, significance, and diagnostic complexities associated with Meckel's diverticulum, given the importance of early and accurate diagnosis for this condition. This approach has the potential to prevent severe complications and facilitate appropriate management for the patient.

This case report discusses a 13-year-old male presenting with GI bleeding, a common manifestation of Meckel's diverticulum, often asymptomatic. Symptoms, when present, typically arise in children under 10 years due to complications. Frequent complications include GI bleeding, abdominal pain, vomiting, distension from obstruction or bowel perforation, and fever from diverticulitis [6,8]. Bleeding results from erosions or ulcers on the adjacent intestinal mucosa due to heterotopic mucosa found in 60% of cases, including gastric mucosa (50%), pancreatic and gastric mucosa (6%), jejunal mucosa (5%), Brunner glands (2%), and gastric and duodenal mucosa (2%) [3,8]. The clinical presentation can mimic other conditions, necessitating a comprehensive preoperative differential diagnosis [8]. Radiology plays a crucial role in aiding the confirmation of the diagnosis, with nuclear radiological examinations being particularly relevant in Meckel's diverticulum cases. A prime instance of a nuclear radiological examination is the Meckel's scan or Technetium-99m pertechnetate scan, which reliably identifies the existence of ectopic gastric mucosa in Meckel's diverticulum. Technetium-99m pertechnetate is intravenously injected and is taken up by both normal mucin-secreting cells and ectopic gastric mucosa, subsequently being secreted into the intestinal lumen [4,7].

Other imaging modalities such as ultrasound and CT scans play crucial roles in the diagnosis of Meckel's diverticulum and its complications. Ultrasound is often used as an initial imaging modality due to its availability, lack of ionizing radiation, and ability to provide real-time imaging. It can demonstrate a blind-ending tubular or sac-like structure arising from the ileum, which may contain echogenic material representing fecaliths or air. However, ultrasound may be limited by operator dependence and difficulty in visualizing the small bowel in obese patients or those with excessive bowel gas [3,6]. On the other hand, CT scans offer detailed cross-sectional imaging of the abdomen and pelvis, allowing for a comprehensive evaluation of the gastrointestinal tract. CT can demonstrate the presence of a Meckel's diverticulum as a blind-ending pouch arising from the distal ileum, often containing air, fluid, or a fecalith. It can also help identify complications such as inflammation, perforation, or abscess formation. However, CT scans expose the patient to ionizing radiation and may require intravenous contrast, limiting their use in certain populations, such as children [2,3].

The patient underwent noncontrast CT scan and the result only showed a thickened intestinal mucosa. As the case involves a pediatric patient with gastrointestinal bleeding of unclear etiology, suspicion arises towards Meckel's diverticulum, where complications manifest as gastrointestinal bleeding in 15%-28% of cases [6]. Meckel's scan using Tc-99m pertechnetate (10 mCi) was subsequently performed dynamically and serially to further confirm the diagnosis. Physiological gastric activity was observed from the first minute, peaking at the tenth minute and persisting throughout. Pathological activity beyond the stomach, coinciding with gastric activity in the right upper hemiabdomen, increased progressively. SPECT/CT confirmation revealed abnormal activity in the right upper hemiabdomen, aligning with the midpole of the right kidney projection to the ileum. Nuclear radiological examinations, such as Meckel's scan or Technetium-99m pertechnetate scans, are appropriate for identifying ectopic gastric mucosa in Meckel's diverticulum. The diagnostic utility is enhanced when the patient is suspected of Meckel's diverticulum without active bleeding. The administered pertechnetate dose (approximately 1.85 MBq/kg or 0.05 mCi/kg) aligns with standard protocols. Post-injection images are captured every 30-60 seconds for 30 minutes. SPECT imaging aids in localizing the diverticulum, and premedication with a proton pump inhibitor reduces gastric mucosa technetium secretion, which increases the radiotracer uptake found in this patient [2,^4^,^7^,9].

The patient underwent laparotomy for ileo-ileal anastomosis resection, revealing a pouch protruding into the lumen identified approximately 50 cm from the ileocecal valve. This intervention is appropriate for symptomatic or complicated Meckel's diverticulum [10]. The diverticulum is consistently located at the antimesenteric border of the distal ileum, with variations in proximity to the ileocecal valve. The diverticulum may also exhibit fibrous layers or adhesions in the umbilical region. The diverticulum's length can reach 15 cm, termed Giant Meckel's diverticulum if the lumen diameter exceeds 5-6 cm. Histopathological examination of Meckel's diverticulum encompasses all layers of the ileal wall. The patient's histology's result confirmed the diagnosis of Meckel's diverticulum with ectopic gastric mucosa. Resected diverticula often exhibit heterotopic tissue (approximately 50%), with gastric mucosa most frequently found (23%-50%). Gastric mucosa types include fundus, antral, and pyloric mucosa. Fundus and corpus mucosa may contain oxyntic glands with parietal and mucosal cells [9,11].

## Conclusion

Meckel's diverticulum is among the most frequent causes of gastrointestinal morbidity in children. Complications are rare but challenging to diagnose. History taking, physical examination, and nuclear imaging can facilitate the identification of the diagnosis, allowing for operative intervention to yield optimal outcomes for the patient. Radiological diagnosis and the selection of imaging modalities for patients with Meckel's diverticulum should consider the symptoms of occurring complications and the patient's age. In this case, the diagnosis of Meckel's diverticulum was established based on the uptake in the right upper hemiabdomen through scintigraphy or Meckel's scan, and SPECT/CT examination provided precise anatomical localization of the abnormal structure with radiotracer accumulation. Surgical measures and histopathological examination have confirmed the presence of Meckel's diverticulum in the patient.

## Patient consent

Written informed consent for the publication of this case report was obtained from the patient.

## CRediT authorship contribution statement

**Damayanti Sekarsari:** Conceptualization, Investigation, Resources, Writing – original draft, Writing – review & editing, Supervision. **Ella Putri Saptari:** Writing – original draft, Writing – review & editing, Investigation. **Mohamad Yanuar Amal:** Writing – review & editing, Supervision. **Quinta Febryani Handoyono:** Writing – review & editing. **Alvita Dewi Siswoyo:** Resources. **Sastiono:** Resources. **Hanifah Oswari:** Resources. **Ening Krisnuhoni:** Resources.
